# Ischemic injury to primary motor and premotor cortices is linked to hyperreflexia in mice

**DOI:** 10.1016/j.nbd.2026.107354

**Published:** 2026-03-14

**Authors:** Sushain Kaul, Lei Liu, Eduardo Candelario-Jalil, Shahabeddin Vahdat

**Affiliations:** a Department of Bioengineering, University of California Riverside, Riverside, CA 92507, USA; b Department of Neuroscience, McKnight Brain Institute, University of Florida, Gainesville, FL 32610, USA; c Graduate Neuroscience Program, University of California Riverside, Riverside, CA 92507, USA

**Keywords:** Ischemic stroke, Hyperreflexia, Spinal cord, Motor cortex, Mice

## Abstract

Post-stroke spasticity (PSS), characterized by hyperreflexia and spastic hypertonia, is a common and debilitating consequence of cerebrovascular injury that impedes motor recovery. Despite its prevalence, the neurophysiological mechanisms underlying PSS remain poorly understood, partly due to the lack of reliable animal models. A known clinical correlate of spasticity is weakened rate-dependent depression (RDD) of the Hoffman reflex (H-reflex), particularly in the upper limbs of chronic stroke patients. However, the mechanistic basis of RDD impairment and its relationship to brain lesion characteristics remain unclear. Here, we characterized the longitudinal progression of H-reflex RDD in a wrist flexor muscle following photothrombotic stroke in mice and examined its association with lesion volume across cortical and subcortical regions using magnetic resonance imaging. RDD was significantly reduced 2–3 weeks after stroke, indicating hyperreflexia. Approximately 60% of stroke mice exhibited weakened RDD. Notably, lesion volumes in contralateral primary (M1) and secondary (M2) motor cortices correlated positively with RDD impairment, linking greater cortical damage to more severe reflex hyperexcitability. These results suggest that damage to motor cortical regions disrupts central reflex-modulating pathways, contributing to post-stroke hyperreflexia. The presented stroke model provides a valuable platform for mechanistic investigations of PSS and for evaluating potential therapeutic interventions.

## 1. Introduction

Stroke is a leading cause of long-term disability worldwide (Martin et al., 2025). Spasticity is a frequent motor complication following stroke, affecting approximately 30–40% of survivors, and is particularly prevalent and functionally disabling in the upper limbs (Ward, 2012; Wissel et al., 2013). It interferes with motor recovery, delays rehabilitation, and contributes to secondary complications including weakness, contractures, and pain (Lundström et al., 2010; Wieters et al., 2021). Clinically, spasticity was defined by Lance as a velocity-dependent increase in tonic stretch reflexes resulting from hyperexcitability of the stretch reflex (Lance, 1990). Although this definition has been refined, the neuroanatomical and pathophysiological mechanisms underlying post-stroke spasticity remain incompletely understood, complicating its precise characterization (Wieters et al., 2021).

Animal models have been instrumental in elucidating mechanisms of neurological disease, yet modeling spasticity has been challenging due to the lack of standardized behavioral and physiological outcome measures. Consequently, reliable mouse models of post-stroke spasticity are scarce. Only a limited number of studies have demonstrated spasticity-like features in mouse stroke models (Isumi et al., 2024; Lee et al., 2014; Toda et al., 2014). Using photothrombotic ischemic lesions targeting the forelimb motor cortex, these studies identified post-stroke impairments in rate-dependent depression (RDD) of the Hoffmann reflex (H-reflex). RDD, defined as the reduction in H-reflex amplitude with increasing stimulation frequency, was diminished following stroke, indicating increased spinal reflex excitability. Clinically, reduced RDD is a hallmark of hyperreflexia and is commonly observed in stroke patients with spasticity (Burke et al., 2013; Lamy et al., 2008). The temporal profile of hyperreflexia following stroke appears broadly comparable between humans and rodent models. In mouse models of focal ischemia, hyperreflexia typically emerges within 1–3 weeks post-stroke (Isumi et al., 2024; Lee et al., 2014; Toda et al., 2014). In contrast, clinical studies in humans report greater variability in onset, with hyperreflexia and spasticity developing as early as approximately 2 weeks and extending up to 3 months after stroke (Nam et al., 2019; Wissel et al., 2010). This wider temporal window in patients likely reflects inter-individual differences in lesion characteristics, severity, and poststroke recovery trajectories.

Recent work using manganese-enhanced MRI demonstrated increased activity in the medullary reticular formation in post-stroke mice exhibiting reduced RDD, implicating brainstem involvement in spastic hyperreflexia (Isumi et al., 2024). Pharmacological evidence further supports the translational relevance of these models, as the anti-spasticity drug baclofen restores attenuated RDD in post-stroke mice (Lee et al., 2014). In contrast to stroke models, genetic and disease-related mouse models of spasticity have more clearly demonstrated underlying mechanisms, particularly implicating impaired glycinergic and GABAergic inhibition leading to motoneuron hyperexcitability (Becker et al., 1986; Biscoe and Fry, 1982).

In humans, H-reflex recordings are widely used to assess spinal motoneuron excitability. Increased H-reflex amplitude and elevated H/M ratios reflect reduced inhibitory control and are characteristic of spasticity following upper motor neuron lesions (Burke, 2016; Hu et al., 2015; Koelman et al., 1993; Little and Halar, 1985; Sheean, 2002). Reduced RDD has likewise been validated as an electrophysiological marker of spastic hyperreflexia and correlates with clinical severity (Lamy et al., 2008; Yang et al., 2015). Neuroimaging studies in stroke patients have further linked spasticity development to lesion location. Lesions involving the internal capsule, basal ganglia, thalamus, corona radiata, and brainstem are consistently associated with increased spasticity risk (Cheung et al., 2016) (Barlow, 2016; Lee et al., 2019; Picelli et al., 2014). Lesion size and involvement of premotor and descending motor pathways further increase this risk (Songjin Ri et al., 2020). Although one study emphasized early clinical severity over lesion location (Volny et al., 2018), the prevailing evidence suggests that damage to descending motor projections is a key determinant of poststroke spasticity (Kuo and Hu, 2018).

Despite these advances, mechanistic links between lesion characteristics and electrophysiological markers of spasticity remain poorly defined, particularly in mouse models. To address this gap, the present study characterizes the temporal progression of H-reflex RDD in a forelimb muscle following ischemic stroke in mice and examines its relationship to lesion volume across cortical and subcortical regions using structural MRI. Using a photothrombotic stroke model targeting the sensorimotor cortex and underlying structures, we tested the hypotheses that (i) RDD reduction emerges with a delayed time course after stroke, and (ii) the magnitude of RDD impairment correlates with lesion volume in cortical regions giving rise to descending motor outputs, including the premotor cortex.

## 2. Materials and methods

### 2.1. Animals

In this study, 25 adult male C57BL/6 J mice (20–30 g, 8–10 weeks old, Jackson Laboratories) were included. They were housed in standard cages with food and water available ad libitum and a 12-h reverse light-dark cycle. All procedures were approved by the University of Florida Institutional Animal Care and Use Committee (IACUC).

### 2.2. Experimental setup and timeline

**Fig. 1.**
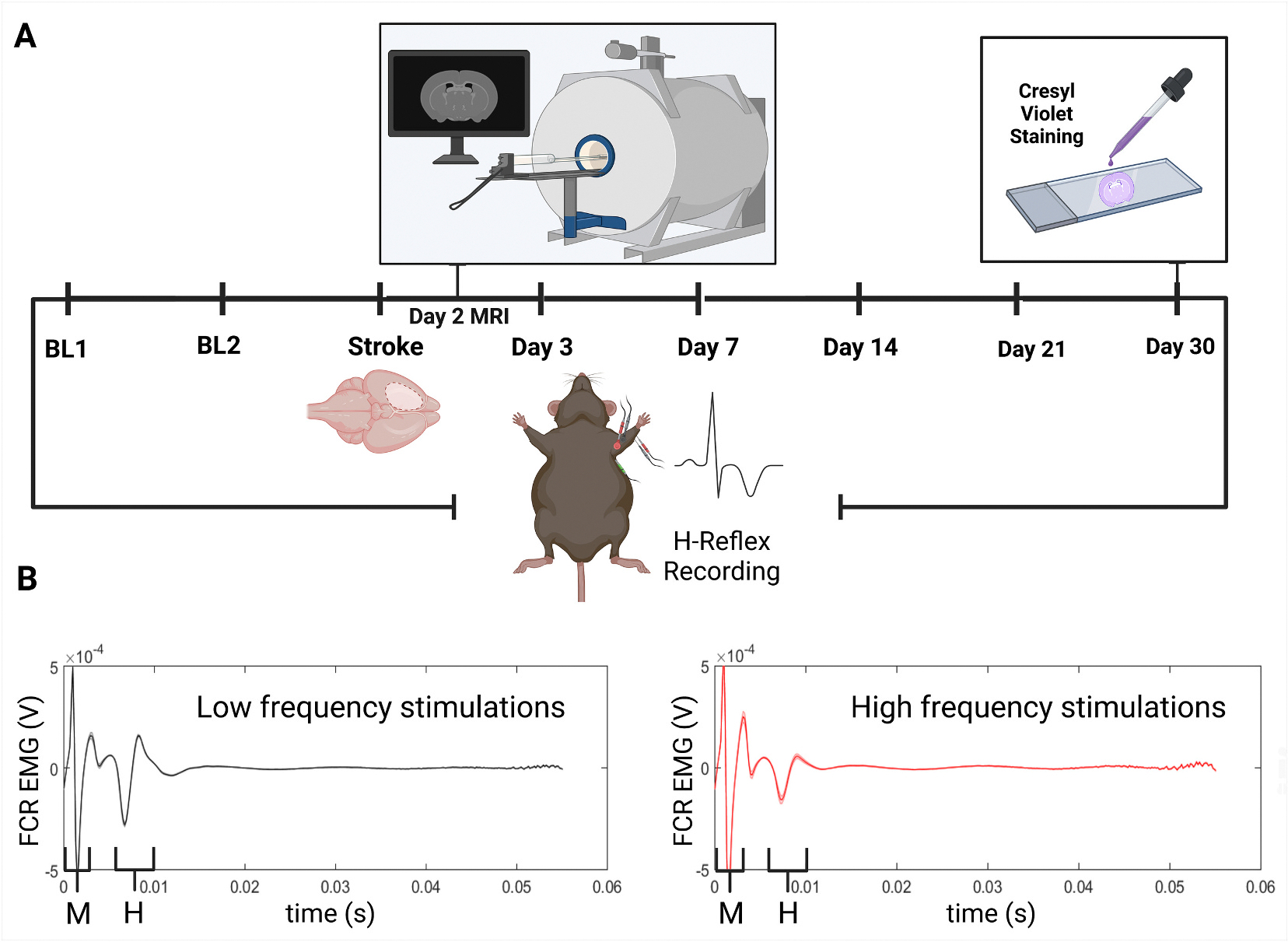
Experimental timeline and H-reflex paradigm. **(A)** Schematic of the experimental timeline. Two baseline H-reflex recordings (BL1 and BL2) were obtained prior to stroke induction. T2-weighted MRI was performed on post-stroke day 2 (PD2). H-reflex recordings were subsequently acquired on PD3, PD7, PD14, PD21, and PD30. Animals were perfused on PD30 for histological assessment using Cresyl violet staining. **(B)** Representative EMG recordings, averaged across trials, illustrating M- and H-wave responses elicited at low (0.1 Hz) and high (3 Hz) stimulation frequencies during the baseline session. The M-wave amplitude remains stable across stimulation frequencies, whereas the H-wave exhibits frequency-dependent depression, with reduced amplitude at higher stimulation frequencies. Shaded area represents the standard error of the mean (SEM) across trials. (For interpretation of the references to colour in this figure legend, the reader is referred to the web version of this article.)

The animals were given one week to acclimatize after arrival before any handling or experiments were done. We assessed the Hoffman reflex (H-reflex) by recording electromyography (EMG) signals from a wrist flexor muscle in response to median nerve stimulation, conducted across multiple days before and after ischemic stroke induction. H-reflex data was collected on two separate pre-stroke baseline days (BL1 and BL2), and then post-stroke days 3 (PD3), 7 (PD7), 14 (PD14), 21 (PD21) and 30 (PD30) as shown in Fig. 1A. A T2-weighted structural MRI scan was also collected on post-stroke day 2 (PD2), giving the mice sufficient time to recover from stroke surgery. H-reflex recordings were performed at the same time of day across all time points. Power analysis based on previous reports indicated that a minimum of 8 animals per group was sufficient to achieve 90% statistical power at an alpha level of 0.05 (Cohen, 1992).

### 2.3. Photothrombotic stroke

A focal ischemic stroke was induced by causing thrombus formation in the microvessels of the targeted brain area. Mice (stroke: 16, sham: 9) were anesthetized with 2% isoflurane diffused in oxygen flowing continuously first in the induction chamber and then through a nose cone as they were placed on a stereotaxic frame. Vital signs, including body temperature, heart rate, and respiration, were continuously monitored. A single dose of buprenorphine sustained release (1.0 mg/kg) was administered subcutaneously before surgery. The surgical area was shaved, and the site was prepared using a scrub of Iodine and 0.9% saline applied 3 times alternately before making a midline incision of the skin over the skull. Bregma was identified on the exposed skull, and the target region for stroke induction was marked to encompass the rostral and caudal regions of the left sensorimotor cortex (+3.0 mm to −2.5 mm anterior-posterior, −3.0 mm to 0.0 mm lateral to bregma). A slit matching the marked area was cut into a piece of aluminum foil and positioned over the skull to restrict light exposure to the targeted brain region. Rose Bengal solution (10 mg diluted in 1 ml of 0.9% saline solution; Sigma-Aldrich, St. Louis, MO, USA) was then injected intraperitoneally (0.12 ml for a 30 g mouse) in the stroke group mice (*n* = 16). After waiting for 5 min, the stroke area was illuminated using a cold light source (50% intensity; CL6000 Zeiss, Oberkochen, Germany) for 15 min. The scalp was then sutured, and mice were allowed to recover with appropriate support. For sham group mice (*n* = 9), 0.9% saline solution was injected intraperitoneally without Rose Bengal, and the remainder of the procedure was conducted identically. Saline was administered following surgery to prevent dehydration.

### 2.4. H-reflex recording

Rate-dependent depression (RDD) of H-reflex was recorded from a wrist flexor muscle using established protocols for forelimb H-reflex measurements in mice (Lee et al., 2014). Mice were first anesthetized using a cocktail of 90 mg/kg ketamine and 10 mg/kg xylazine diluted in 0.9% saline, injected intraperitoneally. Previous works have shown ketamine/xylazine anesthesia to support stable recording conditions for evoked potentials and reflex measures (Zandieh et al., 2003). This is due to the favorable safety profile of ketamine along with the ability of xylazine to counteract ketamine-induced increases in muscle tone, thereby ensuring stable anesthesia during H-reflex recordings and improving measurement consistency (Irwin et al., 2023; Jimenez-Ruiz et al., 2018). Once the mouse was under deep anesthesia (tested with toe pinch), the ventral part of the forelimb was shaved, and the fur was completely cleaned using hair removal cream. The limbs were then stabilized on a clean flat surface using micropore tape. Needle electrodes were used as recording electrodes and were inserted carefully into the flexor carpi radialis (FCR) muscle, with the ground electrode inserted just below the skin near the underarm region. For the stimulation electrodes, we used in-house fabricated stainless steel surface electrodes that were placed over the median nerve between the recording and the ground electrodes and stabilized using electrophysiological gel glue. The positions of the stimulation electrodes were adjusted to elicit robust H-reflex responses, monitored in real time on an oscilloscope. Stimulation intensity was then set between 1 and 3 mA (pulse duration: 200 ms) to evoke an approximately maximum H-reflex amplitude. To calculate RDD, H-reflex responses were recorded at both low and high stimulation frequencies. On each experimental day, mice underwent two sets of trials, each consisting of 20 stimulations at 0.1 Hz (low frequency) and 20 stimulations at 3 Hz (high frequency). The order of low and high frequency stimulation was counterbalanced across the two sets. EMG data was recorded using the TDT LabRat EMG system (Tucker-Davis Technologies) with an amplifier (high-pass filter at 10Hz and low-pass filter at 1000 Hz) paired with TDT Synapse software. Stimulations were done using Aurora Scientific 701C High Power, Biphasic Stimulator integrated with Aurora Scientific DMCv6 LabBook software. The recorded data was then transferred to MATLAB for further analysis.

### 2.5. T2-weighted MRI scanning

T2-weighted MRI brain images of anesthetized mice were acquired on PD2 to assess the lesion volume. All animals in the sham group (*n* = 9) and all but 2 animals in the stroke group (*n* = 14) were imaged due to technical issues in the MRI facility. All MR imaging was conducted using a high field 11.1 T Bruker Scanner housed at the Advanced Magnetic Resonance Imaging and Spectroscopy Facility (AMRIS) within the McKnight Brain Institute at the University of Florida. A standardized scanning setup and imaging protocol were used for all animals, which involved a custom receiver/transmitter MR linear coil designed by MR engineers at AMRIS, which was mounted to a sled designed in-house on which the mouse was also restrained and received continuous Isoflurane anesthesia through a nose cone. The sled also contained ear bars to stabilize the mouse head and minimize any motion artifact. Hot water was circulated in the base of the sled using a tube to maintain the body temperature of the mouse during the scan which was continuously monitored along with the respiration rate.

Each scanning session started with a localizer scan to adjust the head of the mouse to the center of the scanner. Following localizer, a shimming procedure was conducted to ensure uniform distribution of the magnetic field in an ellipsoidal shape covering the brain and the brainstem. After shimming, a high resolution structural T2-weighted MR scan was acquired using a multi-slice T2-weighted fast spin echo sequence signal, with a field of view of 25 × 25 mm, slice thickness of 0.35 mm, repetition time (TR) of 7 s, echo time (TE) of 36 ms, excitation angle of 90 degrees, bandwidth of 100 kHz, with 41 slices and an in-plane resolution of 0.098 × 0.098 mm^2^.

### 2.6. Data analysis

EMG data analysis was performed using MATLAB software. Raw signals were first down-sampled to reduce computational load and then detrended to remove slow baseline drifts. Signals were further band-pass filtered to isolate the relevant frequency components of the M and H waves. For each trial, the latency of the M wave was defined as 0–5 ms and the H wave as 5–12 ms, consistent with previously published data on mouse forelimb M and H wave latencies. (Isumi et al., 2024; Lee et al., 2014). Each recording was inspected manually to adjust for minor variations in latency. At each time point, two trials were collected per frequency for each mouse, with each trial consisting of 20 stimulations. To ensure stability, the first two stimulations of each trial were discarded. The remaining responses were then averaged across the two sets of trials to obtain a representative waveform for each frequency.

The Rate-Dependent Depression (RDD) index was calculated as the ratio of the mean peak-to-peak amplitude of the H-reflex response at high-frequency stimulation (3 Hz) to that at low-frequency stimulation (0.1 Hz). Thus, an RDD index closer to 1 indicates reduced depression (i.e., impaired inhibitory modulation), whereas a lower index reflects stronger rate-dependent depression.

Imaging data analysis was performed using FSL and MATLAB software. All T2-weighted MRI scans underwent brain extraction to remove skull and other tissues from the images and were corrected for bias field. The brain extracted T2-weighted images were then registered to the T2-weighted image of a representative mouse selected as template for group-level analysis using a 12-parameter affine transformation as implemented in FSL. Furthermore, we obtained the registration parameters from the Allen Mouse Brain Atlas (Common Coordinate Framework version 3) (Oh et al., 2014) to the template image, using a 12-parameter affine transformation as implemented in FSL. The mask of each brain area as defined in the Allen Mouse Brain Atlas was then registered to the template space for further region of interest (ROI)-based analysis. While defining the masks, special care was taken to account for any minor midline shifts due to stroke so proper ROIs` are identified. All cortical and subcortical regions that showed overlap with the lesion were selected. Furthermore, lesion masks were manually delineated on the T2-weighted images for each mouse and overall lesion volume was calculated. For each mouse, lesion volume within each ROI was quantified by overlapping the subject-specific lesion mask with the corresponding ROI mask from the Allen atlas, registered to the template. Although some of these ROIs were beyond motor areas, they were still included to account for any potential contributions from these non-motor areas.

### 2.7. Cresyl violet staining

To confirm and visualize the lesion on post-stroke day 30, animals were perfused using 1× PBS followed by 4% Paraformaldehyde Solution in 1× PBS and the brains were extracted. The brains were kept in 30% sucrose solution overnight for cryoprotection. Once the brain sank in the sucrose solution, it was placed in a mouse brain mold, covered with OCT, and frozen at −80 °C until it was sliced. Thirty-micron coronal slices of the brain were made, and the ones with the lesion were used for staining. Cresyl violet stain was used to visualize and differentiate live and dead neurons, thereby identifying the lesion. The stained slices were imaged on a Leica Laser Microdissection scope and visualized using Leica Aperio software. This procedure was performed to confirm the presence and localization of the lesion identified on MRI; it was not used for lesion volume quantification because a complete set of histological sections was not available for all animals.

### 2.8. Statistical and correlation analysis

Statistical analysis was performed using MATLAB and R. Two-factor mixed-effects ANOVA models were constructed with group (stroke vs sham) and time point (BL1, BL2, PD3, PD7, PD14, PD21, PD30) as fixed effects, and RDD ratio as the dependent variable. Post hoc *t*-statistics were performed to determine significant differences in RDD across groups and days using Satterthwaite's method. Prior to performing ANOVA, the assumption of normality for the H-reflex data was evaluated using the Kolmogorov–Smirnov test. To evaluate the relationship between lesion location and hyperreflexia, we calculated linear (Pearson's) and non-parametric (Spearman's) correlations between lesion volume in various brain ROIs and RDD across animals in the stroke group. Given the sensitivity of Pearson's correlation to outliers, we also recalculated correlations after excluding them. Outliers were defined as data points exceeding 2.5 standard deviations from the median total stroke volume.

## 3. Results

### 3.1. RDD of H-reflex following ischemic stroke

**Fig. 2.**
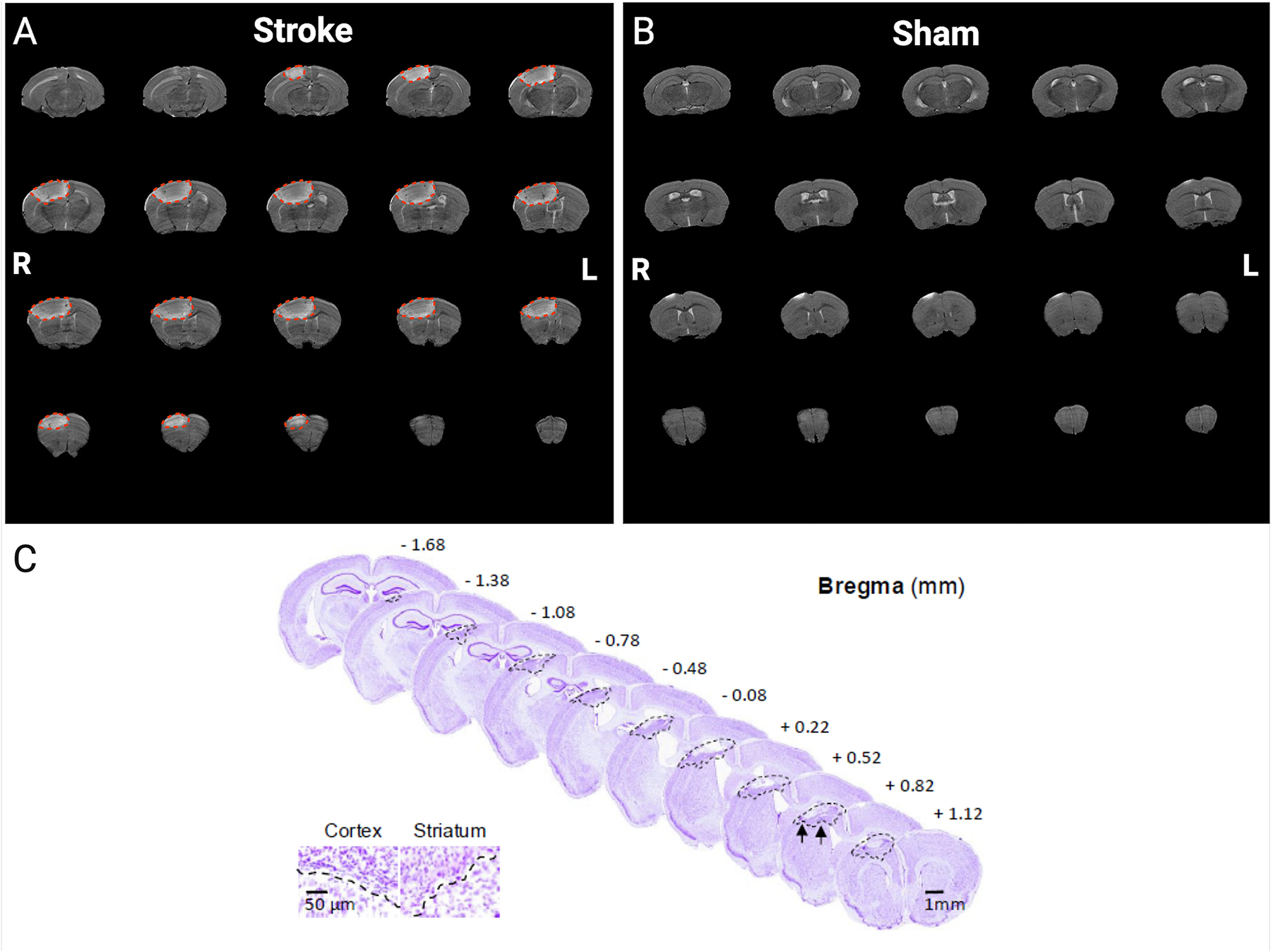
Representative photothrombotic ischemic lesions involving the sensorimotor cortex and underlying subcortical regions. **(A)** Representative T2-weighted MR images of stroke and sham animals acquired on PD2, illustrating lesion location and extent. **(B)** Representative Cresyl violet-stained coronal brain sections obtained on PD30, showing the extent of tissue damage across serial sections. (For interpretation of the references to colour in this figure legend, the reader is referred to the web version of this article.)

Mice were assigned to sham or stroke groups, and the H-reflex from the FCR muscle was recorded at low and high frequencies of median nerve stimulation across multiple days before and after stroke to assess RDD (experimental timeline shown in Fig. 1A). Stroke was induced using a photothrombotic model, resulting in ischemic lesions involving the sensorimotor cortex and underlying subcortical regions. Fig. 2 depicts the lesion area in representative stroke and sham animals based on T2-weighted MRI acquired on post-stroke day 2 (PD2). Lesion location and extent were independently confirmed by Cresyl violet staining of coronal brain sections obtained on PD30.

**Fig. 3.**
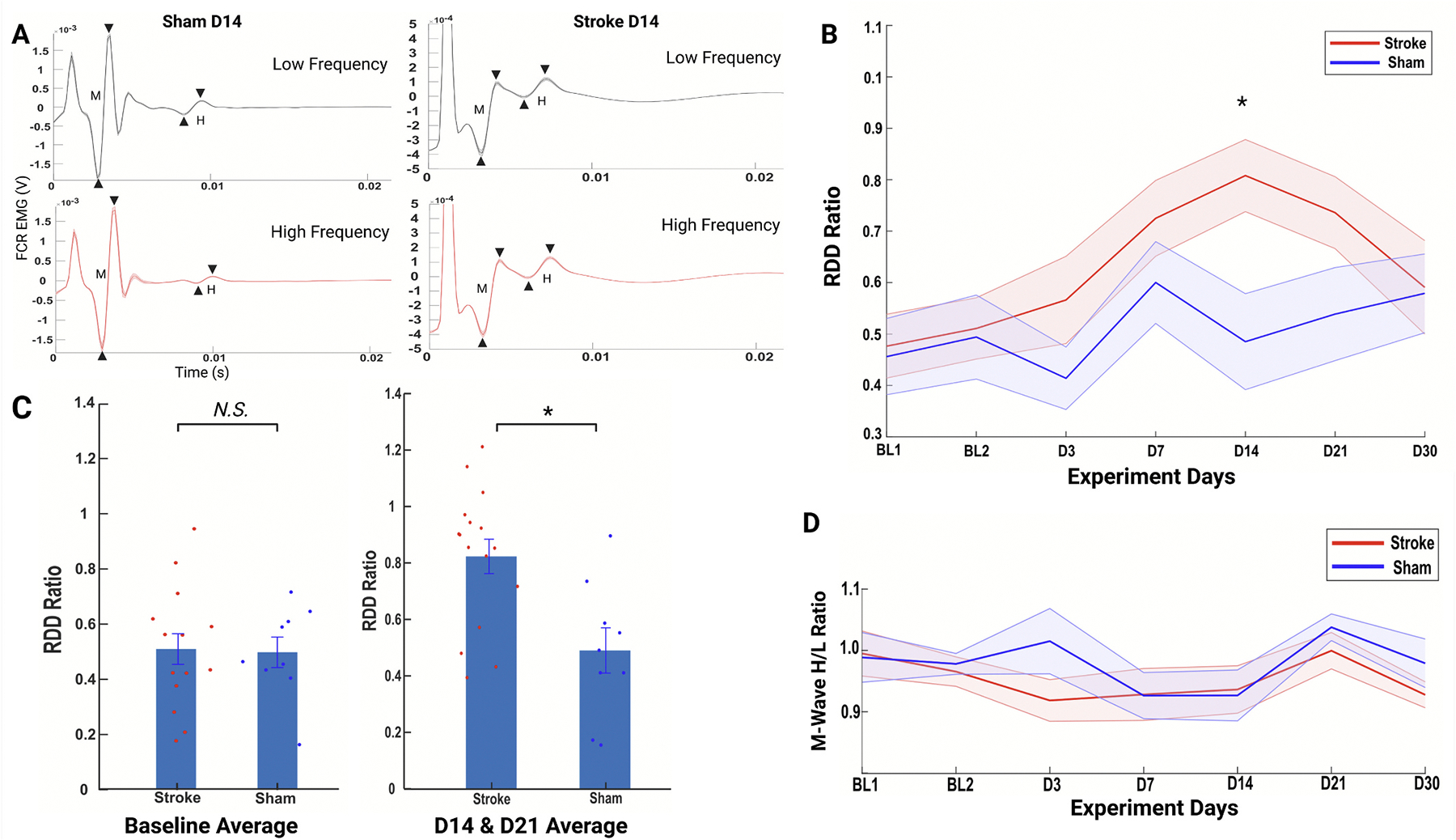
Delayed reduction of H-reflex rate-dependent depression (RDD) following stroke. **(A)** Representative EMG recordings illustrating H-reflex responses elicited at low (black, 0.1 Hz) and high (red, 3 Hz) stimulation frequencies on post-stroke day 14 (PD14) in sham and stroke animals. **(B)** Group-averaged RDD trajectories across experimental time points in stroke (red) and sham (blue) animals. RDD is significantly elevated in the stroke group relative to sham at PD14. **(C)** Summary analysis shows no significant group difference in RDD at baseline (mean of BL1 and BL2), but a significant increase in RDD in the stroke group at 2–3 weeks poststroke (mean of PD14 and PD21). **(D)** Ratios of peak-to-peak M-wave amplitudes at high versus low stimulation frequencies across time points show no significant within- or between-group differences. Shaded regions and error bars indicate the standard error of the mean (SEM). * indicates *p* < 0.05. (For interpretation of the references to colour in this figure legend, the reader is referred to the web version of this article.)

Fig. 1B shows representative H-reflex responses elicited at low and high stimulation frequencies during the pre-stroke baseline session. As expected, the H-wave exhibited frequency-dependent depression, with reduced peak-to-peak amplitudes at higher stimulation frequencies, whereas the M-wave amplitude remained unchanged. Consistent with these observations, Fig. 3B shows that both stroke and sham groups displayed stable and comparable RDD ratios across the two pre-stroke baseline days (BL1 and BL2), confirming the reliability and day-to-day stability of the measurements (stroke group: mean RDD = 0.48 on BL1 and 0.51 on BL2, *t* = 0.53, *p* > 0.5; sham group: mean RDD = 0.46 on BL1 and 0.49 on BL2, *t* = 0.24, *p* > 0.5).

To evaluate post-stroke changes, RDD was measured longitudinally on PD3, PD7, PD14, PD21, and PD30. Representative examples of H- and M-wave recordings at low and high stimulation frequencies on PD14 are shown in Fig. 3A for both stroke and sham groups. Fig. 3B presents the mean RDD ratios across animals for each time point in the stroke and sham groups. Normal distribution of data was confirmed using Kolmogorov-Smirnov test (*p* > 0.1) to ensure the validity of our ANOVA. A mixed-effects two-way ANOVA (group: 2 levels; time point: 7 levels) revealed a significant group × timepoint interaction (*F*_*6, 266.65*_ = 2.52, *p* < 0.05; main effect of Group: *F*_1, 288.44_ = 65.60, *p* < 0.01 and Timepoint: *F*_6, 267.62_ = 2.23, *p* < 0.05), indicating different RDD trajectories in sham versus stroke groups. Between-group comparisons further demonstrated higher RDD in the stroke versus sham group at PD14 (*t* = 2.60, *p* < 0.05, 95% CI [0.064, 0.58]). Subsequent one-way ANOVAs within each group revealed that RDD remained stable in the sham group (*F*_*6*_ = 0.57, *p* > 0.5), but varied significantly in the stroke group across timepoints (*F*_*6*_ = 2.22, *p* < 0.05). Post-hoc comparisons confirmed that RDD in the stroke group increased significantly from baseline (mean of BL1 and BL2) to PD14 (*t* = 3.21, *p* < 0.05, 95% CI [0.09, 0.5]) and PD21 (*t* = 2.51, *p* < 0.05, 95% CI [0.03, 0.48]). No other within-group changes across timepoints were significant (*p* > 0.05). To summarize these effects, we further computed average RDD indices at baseline (mean of BL1 and BL2) and at 2–3 weeks post-stroke (mean of PD14 and PD21). As shown in Fig. 3C, RDD did not differ between groups at baseline (*t* = 0.13, *p* > 0.5), but was significantly elevated in the stroke group compared with the sham group at 2–3 weeks post-stroke (*t* = 3.29, *p* < 0.005, Cohen's d = 1.37, 95% CI [0.12, 0.54]).

To verify the consistency of stimulations and EMG recordings during RDD measurements, we calculated the peak-to-peak ratio of the M-wave at high versus low stimulation frequencies. As shown in Fig. 3D, the M-wave ratio was highly consistent across timepoints and groups (mixed-effects two-way ANOVA: no significant interaction, F_6, 152_ = 0.53, *p* > 0.5; no significant main effects of group F_1, 152_ = 1.58 or timepoint F_6, 152_ = 1.4, both *p* > 0.5). Moreover, the M-wave ratio was not significantly different from 1 at any timepoint in either group (*p* > 0.5).

### 3.2. Assessment of the ischemic volume and its relationship with hyperreflexia

**Fig. 4.**
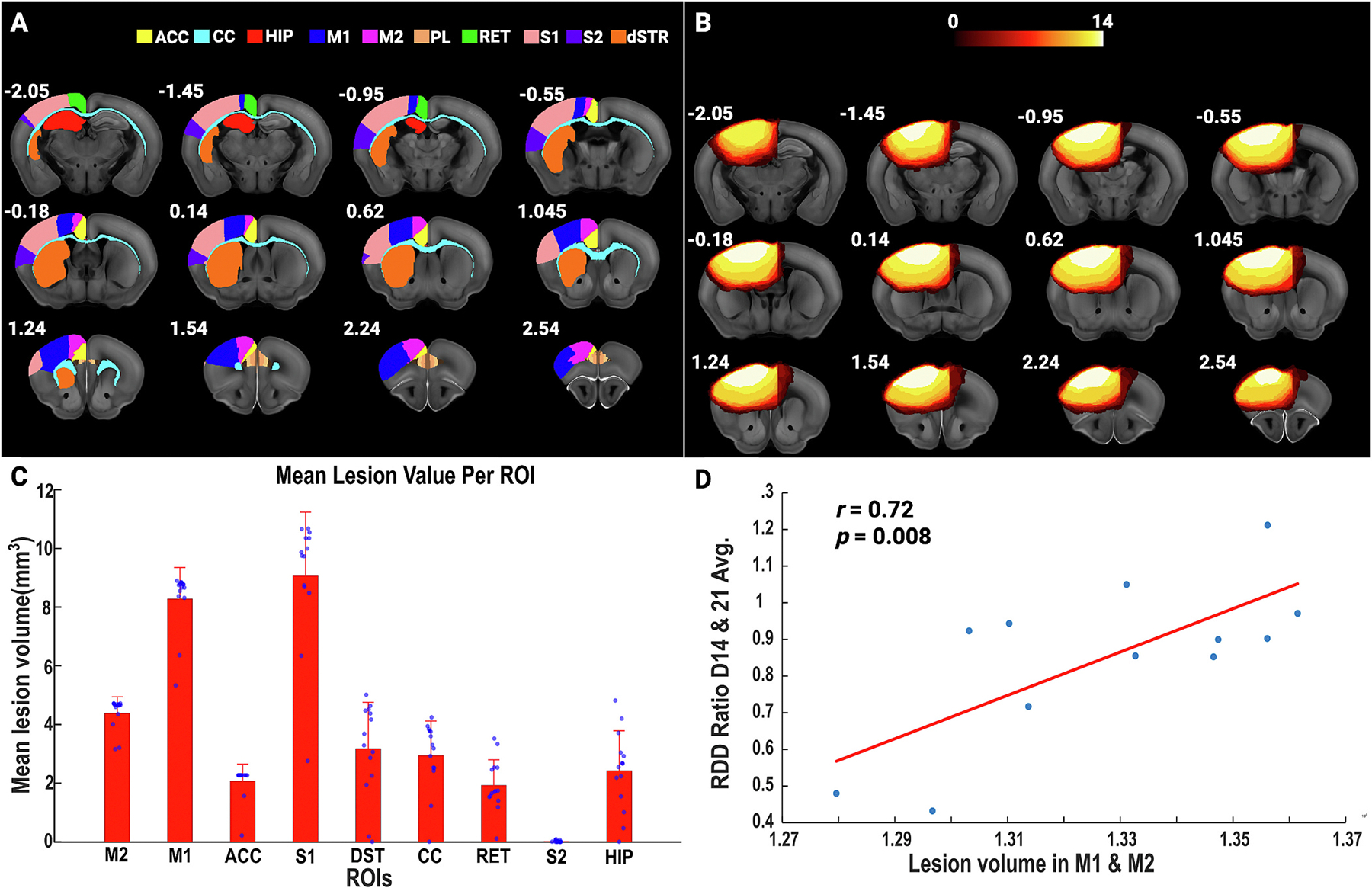
Lesion volume in contralateral M1 and M2 correlates with impaired rate-dependent depression (RDD) after stroke. **(A)** T2-weighted MR image illustrating cortical and subcortical regions of interest (ROIs) affected by stroke, defined using the Allen Mouse Brain Atlas. **(B)** T2-weighted MRI–derived lesion overlap heat map depicting the spatial distribution and extent of lesions across all animals; colour denotes the number of animals (*n* = 14) with a lesion at each voxel. **(C)** Mean lesion volume for each ROI across all mice; M1 and S1 exhibit the largest lesion volumes. **(D)** Total lesion volume in M1 and M2 significantly correlates with RDD impairment at 2–3 weeks post-stroke in the stroke group. Error bars represent the standard deviation. Abbreviations: secondary motor cortex (M2), primary motor cortex (M1), anterior cingulate cortex (ACC), primary somatosensory cortex (S1), dorsal striatum (DST), corpus callosum (CC), retrosplenial cortex (RET), secondary somatosensory cortex (S2), hippocampus (HIP).

Total infarct volume averaged 50.1 mm^3^ (SE = 4.2 mm^3^) across stroke animals, as measured by T2-weighted MRI on PD2. Fig. 4A, B illustrates the regional distribution of infarct likelihood across multiple cortical regions including the primary motor cortex (M1), secondary motor cortex (M2), primary somatosensory cortex (S1), secondary somatosensory cortex (S2), anterior cingulate cortex (ACC), retrosplenial cortex (RET), as well as subcortical regions such as the dorsal striatum (DST), corpus callosum (CC), and hippocampus (HIP). The mean lesion volume across animals within each ROI is presented in Fig. 4C. S1 and M1 exhibited the largest lesion volumes, followed by M2, DST, CC, and HIP.

We next conducted an ROI-based analysis to assess whether the lesion volume in these brain regions correlated with hyperreflexia. This analysis revealed a significant positive correlation between RDD measured 2–3 weeks post-stroke (mean of PD14 and PD21) and lesion volume in M1 (Spearman's ρ = 0.56, *p* = 0.04) and M2 (Spearman's ρ = 0.58, *p* = 0.03) (Supplementary Fig. 1). Moreover, the combined lesion volume of the primary and secondary motor cortices (M1 & M2) exhibited an even stronger association with RDD (Spearman's ρ = 0.67, *p* = 0.01). A significant linear relationship was also observed between RDD and total M1 & M2 lesion volume (Pearson's *r* = 0.72, *p* < 0.01; Fig. 4D). Notably, these relationships remained significant after accounting for two potential outliers. No other region exhibited a significant correlation with RDD, regardless of outlier removal (*p* > 0.05). These results suggest a direct proportional relationship between hyperreflexia and the combined lesion volume of M1 and M2, while RDD shows only a monotonic association with each region individually.

## 4. Discussion

In this study, we demonstrate that ischemic injury to sensorimotor cortical areas induces a delayed reduction in the rate-dependent depression (RDD) of the H-reflex, consistent with the development of hyperreflexia and spasticity-like physiology. Specifically, we observed a significant attenuation of RDD in stroke animals 2–3 weeks after stroke, while sham animals showed stable reflex modulation across all time points. Importantly, we also identified a positive correlation between the extent of lesion volume within the primary (M1) and secondary (M2) motor cortices and the degree of hyperreflexia. This suggests that cortical involvement, particularly in regions giving rise to corticospinal projections, contributes to post-stroke alterations in spinal reflex excitability.

Our findings align with and extend previous work in rodent stroke models that reported reductions in H-reflex RDD following sensorimotor cortex injury (Lee et al., 2014; Toda et al., 2014). A recent study using a comparable mouse photothrombotic stroke model reported an early attenuation of the H-reflex at 2–3 days post-stroke (Isumi et al., 2024), whereas other studies employing the same model have observed this attenuation at later time points (14–21 days post-stroke) (Lee et al., 2014; Toda et al., 2014), consistent with our results. By incorporating MRI-based lesion mapping, we showed that the extent of damage within M1 and M2 correlates with electrophysiological impairment. These findings support the hypothesis that cortical lesions influence spinal reflex circuitry either directly, through corticospinal projections, or indirectly, via descending modulation of brainstem motor centers. Consistent with a role for the brainstem in spasticity, a recent study demonstrated increased neural activity in the ventral medullary reticular formation following a comparable photothrombotic stroke model in mice that developed hyperreflexia (Isumi et al., 2024). Additional mechanisms may involve altered spinal inhibitory neurotransmission leading to increased motoneuron excitability (Toda et al., 2014). The late-onset impairment of spinal reflex pathways further suggests that these changes may be mediated by secondary neurodegeneration or by the loss of descending inputs to brainstem and/or spinal targets. Future studies will be necessary to disentangle these mechanisms and to determine their relative contributions to spinal circuit reorganization after stroke.

The observed results mirror human neuroimaging studies linking spasticity to lesion overlap in the internal capsule, premotor cortex, and basal ganglia (Barlow, 2016; Daniel K Cheung et al., 2016; Kyoung Bo Lee et al., 2019), reinforcing the translational relevance of our model. The convergence of rodent and human evidence highlights the primary motor and premotor cortices as key substrates for spasticity development after stroke. Furthermore, we assessed the H-reflex using surface electrodes, in contrast to needle electrodes used in previous studies (Lee et al., 2014; Toda et al., 2014), to minimize muscle trauma and allow repeated longitudinal measurements.

The delayed onset of hyperreflexia we observed is also notable. While acute injury induced robust infarcts by post-stroke day 2, hyperreflexia did not emerge until 2–3 weeks post-stroke. This temporal dissociation suggests that post-stroke spasticity may not be a direct consequence of lesion size alone, but rather involves progressive maladaptive plasticity in spinal and supraspinal circuits. Such plasticity may include reduced GABAergic and glycinergic inhibition at the spinal level, as suggested by genetic models of spasticity (Becker et al., 1986; Biscoe and Fry, 1982; Bose et al., 2015; Biscoe et al., 1984), or compensatory hyperexcitability within brainstem motor pathways, as previously shown in medullary reticular formation (Isumi et al., 2024). Our findings, therefore, support a two-stage model in which cortical injury sets the stage for secondary network adaptations that culminate in spastic hyperreflexia.

Clinically, the impaired RDD observed in our model closely parallels electrophysiological findings in stroke patients, where reduced reflex depression and elevated H/M ratios correlate with clinical measures of spasticity (Aymard et al., 2000; Burke, 2016; Hu et al., 2015; Koelman et al., 1993; Lamy et al., 2008). This strengthens the utility of our model for preclinical testing of anti-spasticity interventions. Pharmacological agents such as baclofen have been shown to normalize RDD in both patients and rodents (Lee et al., 2014; Meythaler et al., 2001). Future work could employ this model to test new therapeutic strategies, including neuromodulatory or rehabilitative interventions targeting corticospinal and brainstem-spinal pathways.

Several limitations should be acknowledged in our current study. First, while our correlation analysis implicates M1 and M2 lesion volume, we cannot rule out contributions from other regions, including subcortical and brainstem structures, which were variably affected in our model. Second, electrophysiological assessments were performed under anesthesia, which may modulate reflex excitability; complementary awake assessments could strengthen translational relevance. Third, our study focused on forelimb reflexes; whether similar dynamics occur in hindlimb circuits remains to be determined. Additionally, the lack of any motor assessment related to post-stroke spasticity limits our study to only the electrophysiological aspect of spasticity. Finally, the relatively small sample size may have limited the detection of weaker correlations with other regions.

In conclusion, we provide evidence that ischemic injury to the motor cortex in mice is linked to delayed hyperreflexia, as indexed by reduced H-reflex RDD, and that the magnitude of this impairment correlates with lesion volume in M1 and M2. These findings establish a clinically relevant mouse model of post-stroke hyperreflexia, underscore the central role of cortical motor areas, and open avenues for mechanistic and therapeutic studies targeting maladaptive plasticity in descending motor pathways.

## Supplementary Material

MMC1

Supplementary data to this article can be found online at https://doi.org/10.1016/j.nbd.2026.107354.

## Data Availability

Data will be made available on request.
