# Supplementary figures and images for: Ischemic injury to primary motor and premotor cortices is linked to hyperreflexia in mice
Source: Neurobiol Dis. Author manuscript; Available in PMC 2026 Jul 6. (PMC13335741; doi:10.1016/j.nbd.2026.107354)

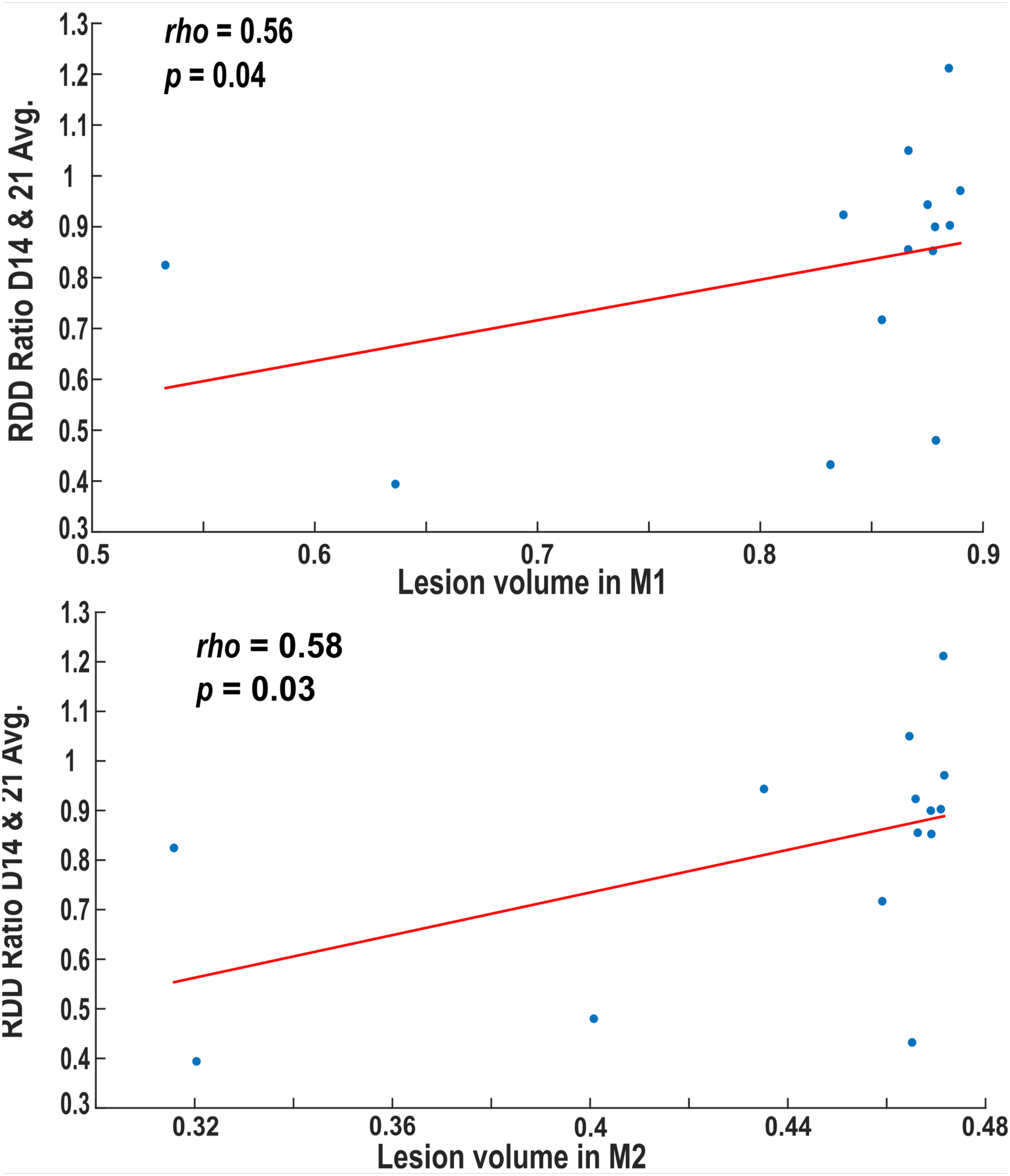

Supplement: MMC1 [file NIHMS2181364-supplement-MMC1.jpg]
